# Acupuncture for the Treatment of Taxane-Induced Peripheral Neuropathy in Breast Cancer Patients: A Pilot Trial

**DOI:** 10.1155/2018/5367014

**Published:** 2018-10-21

**Authors:** Young Ju Jeong, Min Ah Kwak, Jung Chul Seo, Seong Hoon Park, Jin Gu Bong, Im Hee Shin, Sung Hwan Park

**Affiliations:** ^1^Department of Surgery, School of Medicine, Catholic University of Daegu, Daegu, Republic of Korea; ^2^Department of Oriental Internal Medicine, College of Oriental Medicine, Daegu Haany University, Daegu, Republic of Korea; ^3^Comprehensive and Integrative Medicine Institute, Daegu, Republic of Korea; ^4^Department of Surgery, Raphael Hospital, Daegu, Republic of Korea; ^5^Department of Medical Statistics, School of Medicine, Catholic University of Daegu, Daegu, Republic of Korea

## Abstract

**Objectives:**

Some chemotherapy drugs can damage the nerves and cause peripheral neuropathy which is accompanied by severe neuropathic pain or gait impairment. The purpose of this study was to assess the feasibility and the safety of acupuncture for the treatment of peripheral neuropathy following chemotherapy in Korean breast cancer patients.

**Design:**

This study was a prospective single-arm observational study using before and after measurements in breast cancer patients presenting with taxane-induced peripheral neuropathy.

**Settings/Location:**

This study was performed at East-West Medical Center at Daegu Catholic University Hospital, Daegu, South Korea.

**Interventions:**

Acupuncture was administered 3 times a week for 4 consecutive weeks, for 25 ± 5 minutes at each session.

**Outcome Measures:**

The primary outcome measure was severity of CIPN using the Neuropathic Pain Symptom Inventory (NPSI) assessed by a self-administered questionnaire and Nerve Conduction Study (NCS) of extremities. The secondary outcome measure was quality of life (QoL) assessed by a self-administered questionnaire using the 36-Item Short From Health Survey (SF-36).

**Results:**

Acupuncture significantly reduced the severity of CIPN assessed by NPSI score. Four weeks after the last treatment, the symptoms were not aggravated. According to NCS, 42.9% of participants showed improvement of sensory neuropathy. At the end of the treatment, SF-36 scores were significantly increased for variables including physical functioning, role limitations due to physical health problems, social functioning, and general health perceptions compared to those of baseline measurement.

**Conclusions:**

Acupuncture improved symptoms of CIPN and QoL in Korean women suffering from peripheral neuropathy after chemotherapy using taxane for breast cancer. The effects of acupuncture lasted for at least 1 month after the treatment.

## 1. Introduction

Breast cancer is the second most common cancer in the world as well as in Korean women [1, 2]. With an increase of active breast cancer screening and improvement in treatment, early detection and treatment of breast cancer have contributed to reduced breast cancer mortality, but breast cancer is still the leading cause of cancer-related death [3]. Treatments for breast cancer depend on the type and stage of breast cancer and conventional treatment includes combination of surgery, radiotherapy, chemotherapy, endocrine therapy, and targeted therapy. For decades, many effective drugs for breast cancer have been developed and for chemotherapy, taxanes such as paclitaxel and docetaxel have remained standard therapeutic agent in breast cancer [4]. As with most chemotherapeutic drugs, taxanes attack cancer cells but also affect healthy cells and can cause a number of side effects. One of the side effects of taxane is peripheral neuropathy.

Chemotherapy-induced peripheral neuropathy (CIPN) is a neuropathic disorder caused by neurotoxic chemotherapeutic agents including taxanes [5]. CIPN is a dose-limiting side effect and severe symptoms can lead to a dose reduction and/or early discontinuation of chemotherapy [6, 7]. Also, CIPN can severely impair quality of life (QoL). However, although several promising methods including some natural products and complementary therapies have been suggested [8], there are no definite treatments for CIPN yet [7, 9].

Previous studies have suggested that acupuncture is effective in reducing peripheral neuropathy caused by other primary disease [10] and CIPN among patients with various cancer types [11–13]. Schroeder et al. [13] showed a positive effect of acupuncture on CIPN in patients who underwent chemotherapy with different regimens for the treatment of colon cancer, bronchial cancer, lymphoma, and breast cancer. However, there are limited reports focusing on breast cancer among studies on the effects of acupuncture for treatment of CIPN and there are no available data in Korean breast cancer patients.

In recent years, acupuncture is increasingly being integrated into adjuvant treatment of cancer patients [14], but the protocol of acupuncture is not standardized and the treatment outcomes are inconsistent [15]. To utilize acupuncture for the treatment of CIPN in breast cancer based on evidence-based medicine, more data on clinical trials are necessary. In this study, we aimed to investigate the effects and safety of acupuncture for treatment of CIPN in Korean breast cancer patients. We also evaluated QoL of the patients suffering from CIPN. As far as we know, this is the first prospective study to show the data on the effects of acupuncture on the treatment of taxane-induced peripheral neuropathy and QoL in breast cancer patients with CIPN in Korea.

## 2. Materials and Methods

### 2.1. Study Design

**Figure 1:**
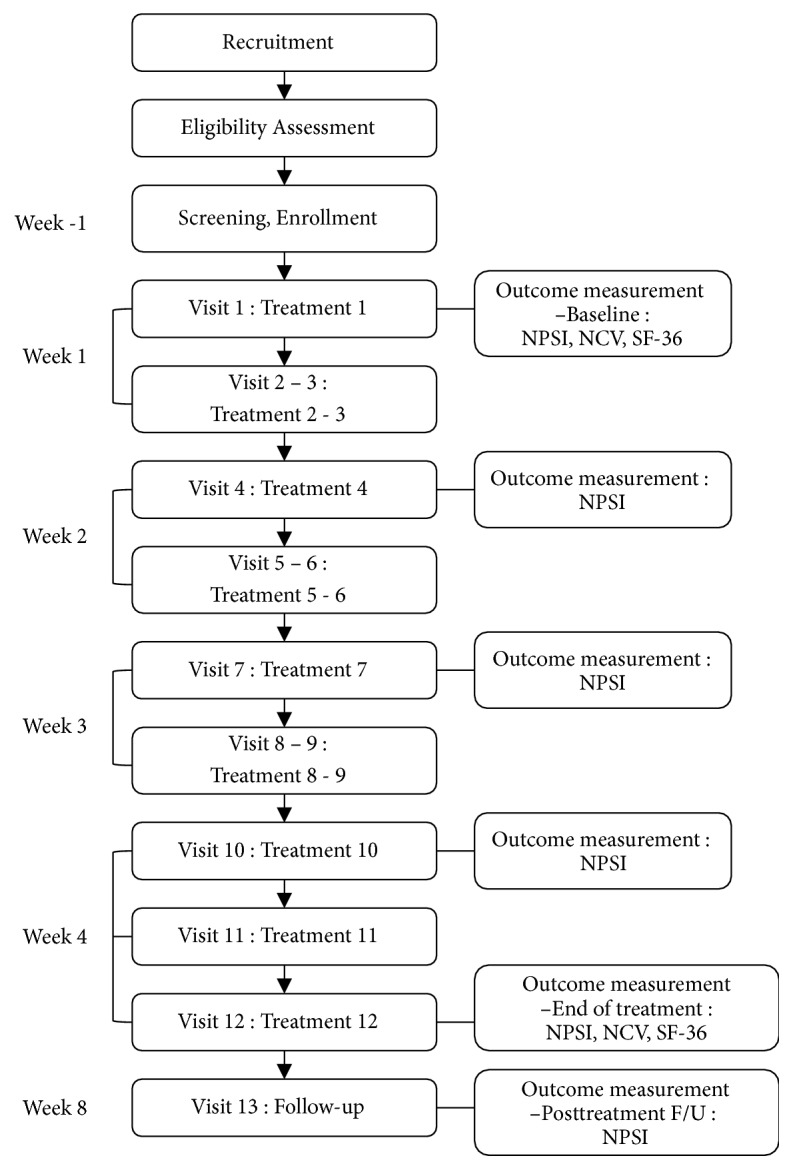
A schema of the study visits and measurements.

This was a prospective, single-arm, pilot trial and an observational study using before and after measurements. Participants received 12 sessions of acupuncture, delivered 3 times a week for 4 consecutive weeks. The primary and secondary outcomes of the trial were the severity of CIPN and QoL. Participants were evaluated for the outcome measurements 6 times in total: once before acupuncture treatment begins (baseline), once a week during treatment for the next 3 weeks, at the end of the treatment, and 4 weeks after the final treatment. A schema of the study schedule is shown in Figure 1. Ethical approval for the study was obtained from the Institutional Review Board of Daegu Catholic University Hospital.

### 2.2. Study Participants

The participants were recruited for the trial from August 2013 through December 2013. Written informed consent was obtained from all participants before enrollment according to the study protocol. After eligible patients were screened, participants were enrolled.

The inclusion criteria were as follows: (1) patients with symptoms of peripheral neuropathy caused by chemotherapy for the treatment of breast cancer; (2) patients with grade I or higher grade of CIPN according to the definition of the World Health Organization (WHO) grading system [16]; (3) voluntary participation; (4) those who can follow-up during the study period; (5) cessation of other pharmacologic or alternative treatments for CIPN during the study period; (6) no drinking of alcohol during the study period. The exclusion criteria were as follows: (1) pharmacologic or other alternative treatment for peripheral neuropathy during the trial; (2) peripheral neuropathy caused by other disease; (3) patients with progressive or metastatic breast cancer; (4) serious medical or psychiatric conditions that made the patient unsuitable to participate in the trial.

### 2.3. Interventions

The study protocol consisted of an eight-week process including 4 weeks of acupuncture treatment and 4 weeks of follow-up after the last treatment. Acupuncture was administered 3 times a week during the first 4 consecutive weeks. For all acupuncture sessions, vital signs including blood pressure, pulse rate, and body temperature were evaluated at each session before the acupuncture treatment.

**Figure 2:**
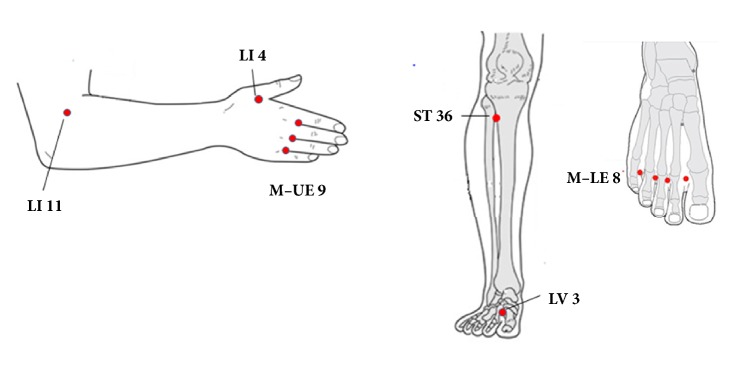
Anatomical position of acupuncture points prescriptions.

Acupuncture was performed by Traditional Korean Medicine physicians who were registered with the government and were specialists in acupuncture treatment. Acupuncture points were referenced to a standard acupuncture textbook [17] and selected based on the results of previous studies [12, 13, 18]. A total of 6 acupuncture points were selected: LI 4, LI 11, ST 36, LV 3, M-UE-9 (*Ba Xie*), and M-LE-8 (*Ba Feng*). The anatomical positions of the acupuncture points were shown in Table 1 and Figure 2, which was referenced to the Points Acupuncture References Software (https://www.points-pc.com). To avoid complications of needling extremity such as infection or exacerbating lymphedema on the affected ipsilateral upper extremity, acupuncture points were limited on the opposite upper extremity of the affected breast and both lower extremities.

A total of 18 needles were used in each acupuncture treatment session. The acupuncture needles were sterile, disposable stainless steel needles (40 × 0.2 mm; Dongbang Acupuncture Inc., Chungcheongnam-do, Korea). Before acupuncture, the skin was swabbed with an alcohol prep pad. Acupuncture treatments took 25 ± 5 minutes at each session. For acupuncture, needles were inserted 5-10 mm deep into the skin of the selected acupuncture points and were gently manipulated manually to obtain De Qi, that is, a needle sensation referring to pain, numbness, and distension felt around the point after the needle is inserted to a certain depth as well as the operator's sensation of tension around the needle [19]. After 10 minutes of needle insertion, the needle positions were controlled by gentle rotation without evoking needle sensation.

### 2.4. Study Outcomes

The primary outcome measure was severity of CIPN using combined 2 outcomes assessed by the Neuropathic Pain Symptom Inventory (NPSI) and Nerve Conduction Study (NCS). The secondary outcome measure was QoL assessed by a self-administered questionnaire using the 36-Item Short From Health Survey (SF-36). The NPSI was assessed 6 times in total during the study period, and NCS and the SF-36 were evaluated once before acupuncture treatment begins (baseline) and at the end of the treatment.

The NPSI is a self-administered questionnaire to evaluate the different symptoms of neuropathic pain [20]. It consists of 10 descriptors reflecting five distinct dimensions of neuropathic pain and 2 items for assessing the duration of spontaneous ongoing and paroxysmal pain. The descriptors include 1 descriptor for burning (superficial) spontaneous pain, 2 for pressing (deep) spontaneous pain, 2 for paroxysmal pain, 3 for evoked pain, and 2 for dysesthesia/paresthesia. Each of these descriptors during the last 24 hours is quantified on a 0-10 numerical scale, in which 0 was ‘no pain' and 10 was ‘the most intense pain imaginable' [20]. A total intensity score was calculated as the sum of the scores of the 10 descriptors.

For objective measurements, NCS was done with a Medelec Synergy electromyography machine (Oxford Instrument Medical Ltd., Surrey, UK) according to the standard nerve conduction techniques [21]. Nerve conduction velocity measurements were performed on the median nerve and the ulnar nerve of upper extremity and the peroneal nerve, the tibial nerve, and the sural nerve of both lower extremities. The amplitude of the motor and sensory nerve responses was measured, and F-wave study and H-reflex study were done. All studies were performed at room temperature.

For a secondary measurement, the SF-36 questionnaire was used to evaluate QoL of the participants. The SF-36 is a 36-item, patient-reported survey of health status and commonly used to measure QoL [22]. The SF-36 consists of eight health concepts, including physical functioning, role limitations due to physical health problems, role limitations due to personal or emotional problems, vitality (energy and fatigue), emotional well-being (general mental health), social functioning, bodily pain, and general health perceptions. All responses were scored according to the scoring rules version 1.0 [23].

### 2.5. Statistical Analysis

Participants characteristics were evaluated using descriptive analysis with mean value and standard deviation for continuous outcomes, and frequency and percentage for categorical data. To investigate changes in the severity of CIPN from baseline to 4 weeks after the final acupuncture treatment, a repeated-measures analysis of variance using the NPSI was performed. Changes in NCS measurements and the SF-36 scores across all measurement points were analyzed using paired t-test or Wilcoxon signed rank test for continuous outcomes. Other variables were analyzed using paired t-test or Wilcoxon signed rank test for continuous data and Friedman test for categorical data. Data was analyzed using the SPSS version 19.0 (SPSS Inc., Chicago, IL). A* p*-value of < 0.05 was considered statistically significant. In principle, intention-to-treat analysis was applied first, and intention-to-treat analysis and per protocol analysis were done in all statistical analyses.

## 3. Results

### 3.1. Participant Flow

A total of 10 Korean women with breast cancer who were suffering from CIPN were enrolled in this trial. All participants completed the full course of 12 acupuncture treatments. All participants provided data for the primary end point and compliance was good. The participants followed the study schedule shown in Figure 1 according to the study protocol.

### 3.2. Baseline Characteristics

The mean age at baseline was 58.7 ± 7.5 years (range, 45–67). All participants have received adjuvant chemotherapy with the anthracycline followed by taxane-based regimen. The mean time from the end of chemotherapy to the start of acupuncture treatment was 6.2 months. Five out of 10 participants were under endocrine therapy with tamoxifen, letrozole, or anastrozole at the time of enrollment. Eight participants had comorbidity but declared no neuropathic symptoms before chemotherapy. No participant reported the use of dietary supplements to relieve peripheral neuropathy at baseline or during the entire study period. The demographic and clinical characteristics of the participants are shown in Table 2. All patients presented several symptoms of peripheral neuropathy including burning, pressure, squeezing, electric shocks, stabbing, pins and needles, and tingling.

### 3.3. Effects of Acupuncture on Severity of CIPN Assessed Using the NPSI

**Figure 3:**
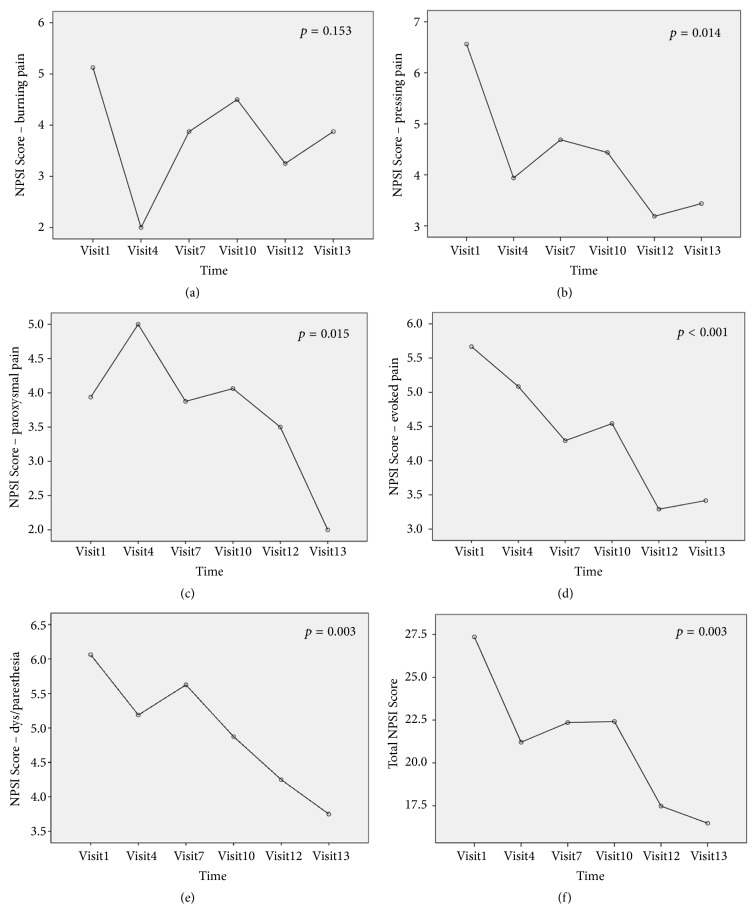
Changes in mean scores of the Neuropathic Pain Symptom Inventory (NPSI) over time: (a) mean NPSI score for burning pain, (b) pressing pain, (c) paroxysmal pain, (d) evoked pain, (e) dysesthesia/paresthesia, and (f) total NPSI score.

The primary endpoint was severity of CIPN using combined 2 endpoints, the NPSI, and NCS. Total NPSI score for all participants was significantly reduced at the end of treatment (*p* = 0.003). In subgroup analysis for distinct dimensions of neuropathic pain, spontaneous pressing pain, spontaneous paroxysmal pain, evoked pain, and dysesthesia/paresthesia were significantly improved (*p* = 0.014,* p* = 0.015,* p* < 0.001, and* p* = 0.003, respectively). Changes in mean NPSI score from baseline to 4 weeks after the final acupuncture session are shown in Figure 3. The effects of acupuncture on the NPSI score persisted at 4 weeks after the last acupuncture session.

### 3.4. Effects of Acupuncture on Severity of CIPN Assessed by NCS

At baseline, 3 out of 10 participants showed no definite electro-physiologic evidence of neuropathy in NCS. Among 7 participants with abnormal NCS, 3 participants (42.9%) showed improvement of NCS at the end of the treatment, while 4 (57.1%) remained unchanged (Table 3). In the analysis of the NCS measurement of each nerve for all participants, there was no significant change from the baseline to the end of the acupuncture treatment.

### 3.5. Effects of Acupuncture on QoL of Breast Cancer Patients with CIPN Assessed by the SF-36

We evaluated QoL of the participants using the SF-36 questionnaire. Among health concepts of the questionnaire, physical functioning, role limitations due to physical health problems, social functioning, and general health perceptions were significantly improved at the end of the acupuncture treatment (*p* = 0.043,* p* = 0.008,* p* = 0.007, and* p* = 0.003, respectively) (Table 4).

### 3.6. Safety

All participants were well adapted to acupuncture treatment during the entire treatment period. No serious adverse events were reported.

## 4. Discussion

In this study, acupuncture reduced symptoms of CIPN in Korean breast cancer patients. Also, acupuncture improved QoL of these patients. The effects of acupuncture lasted for at least 1 month after the acupuncture treatment. Our results suggest that acupuncture is a useful alternative to relieve symptoms of CIPN in breast cancer patients suffering from peripheral neuropathy after chemotherapy. In addition, acupuncture treatment can provide additional benefits for breast cancer survivors by improving QoL.

It is important to recognize that our study focused on the CIPN caused by taxane in breast cancer. Since the effect of acupuncture on pain due to peripheral neuropathy had been reported [10, 24], there have been efforts to apply acupuncture to the treatment of CIPN in cancer survivors [8, 11–13, 18, 25]. Several studies showed that acupuncture improved CIPN [12, 13], but these studies included patients with CIPN caused by different chemotherapeutic agents in patients with a variety of malignancies. In breast cancer, Ben-Horin et al. [26] showed that a joint protocol of acupuncture and reflexology improved symptoms of CIPN, but this was a retrospective series and the chemotherapy regimens varied. Xiong et al. [27] reported clinical efficacy of acupoint injection for CIPN in patients with breast cancer, but the authors also included other neurotoxic regimens besides taxane. Although many chemotherapeutic agents including platinum agents, taxanes, vinca alkaloids, thalidomide, and bortezomib can cause CIPN, pathophysiologic process is complicated and depends on the type of drug [28]. Recently, Ventzel et al. [29] showed that characteristics of CIPN were different between oxaliplatin and docetaxel treatment group, and the results suggest different pathophysiology of CIPN depending on the chemotherapeutic agents. In breast cancer, taxane-based regimens are commonly used for chemotherapy and can cause CIPN [4]. Taxane-induced neurotoxicity has been demonstrated as a result of axonal degeneration secondary to loss of depolymerization of the microtubule, mitochondrial damage and other potential mechanisms [28]. As neuropathic pathways and characteristics of CIPN are drug-specific, the effect of acupuncture on CIPN can vary depending on the chemotherapeutic agent a patient is treated with. Our study focused on taxane-induced peripheral neuropathy in breast cancer. Our results add new data to the existing literature and support the feasibility of acupuncture for the treatment of taxane-induced peripheral neuropathy in breast cancer.

Acupuncture has been used for the treatment of CIPN as one of adjunctive management in integrative cancer care [15]. Nevertheless, there is no consensus on the choice of acupuncture point and the protocol of acupuncture treatment. In a survey of acupuncture and oriental medicine practitioners [15], needle retention times and treatment frequency varied among the practitioners. In other previous studies, duration of intervention varied from 30 days to 28 weeks [11–13, 18, 27, 30]. Acupuncture points were varied among the studies and the most commonly used points were LI 4, LI 11, SP 6, ST 36, LV3,* Ba Xie*, and* Ba Feng*.  Table 5 compares the studies involving acupuncture for CIPN. Notable is that acupuncture was effective in CIPN despite the differences in protocol. In our study, we tried to standardize the protocol of acupuncture treatment for the treatment of CIPN and set up the protocol that includes a total of 12 acupuncture sessions for 4 consecutive weeks. We chose acupuncture points based on the meridian theory and the selected points was 6 out of the commonly used acupuncture points. Our results showed that acupuncture treatment with a relatively short period of 4 weeks was effective in the treatment of CIPN. Also, common acupuncture points that are not individualized to each patient's symptoms were useful. Our findings suggest that it is feasible to develop a standardized protocol for applying acupuncture treatment to the treatment of CIPN. A standardized protocol of acupuncture treatment for CIPN would be useful in clinical practice. Currently, there are several ongoing clinical trials evaluating the role of acupuncture or electroacupuncture for the treatment of CIPN in breast cancer patients (NCT02129686, NCT02615678, KCT0000506 [31]). According to the results of our study and other clinical trials, a new standardized and clinically available protocol of acupuncture treatment for CIPN treatment in breast cancer could be established.

One of the strengths of our study is the use of validated outcome measurements. In this study, combined 2 endpoints, the NPSI and NCS, were used as primary endpoint. We used the NPSI to evaluate patient's symptom severity of peripheral neuropathy, whereas most of previous studies measured symptom severity subjectively [11, 12, 18, 26, 30]. The NPSI is a self-questionnaire designed to evaluate the different symptoms of neuropathic pain and was validated to allow discrimination and quantification of distinct clinically relevant dimensions of neuropathic pain [20]. In this study, acupuncture treatment improved peripheral neuropathy when assessed by total NPSI score, especially spontaneous pressing pain, spontaneous paroxysmal pain, evoked pain, and dysesthesia/paresthesia. Interestingly, burning pain was improved at first but relapsed early, indicating that burning pain is involved different pathophysiological pathway such as nociceptive pathway damage whereas spontaneous paroxysmal pain is related to the nonnociceptive, large-myelinated fibers [32]. Also, we used NCS for objective outcome measure. The use of NCS can provide reliable data and comparable results for studies using acupuncture in the management of CIPN. Previous studies showed improvement in NCS after acupuncture treatment [13, 27], but in this study, only 42.9% of participants showed significant improvement in NCS after acupuncture treatment. Our results could be due to the relatively short interval between baseline NCS measure and follow-up at 4 weeks, whereas Schroeder et al. [13] performed NCS before acupuncture treatment and again 6 months later. Long-term follow-up NCS measure will be needed to clarify the effect of acupuncture on NCS. Furthermore, we analyzed the effects of acupuncture on QoL using the SF-36. Although CIPN is likely to impair QoL of the patients with CIPN [33], only a few studies evaluated QoL as an outcome on the effects of acupuncture on CIPN [11, 25, 31]. The SF-36 is validated self-administered questionnaire of health and commonly used in assessing QoL [34]. Our results with improved the SF-36 scores showed the impact of acupuncture on QoL in patients with CIPN.

Our study had several limitations including small sample size and nonrandomized, single-arm study without control group. Despite these limitations, to the best of our knowledge, this is the first prospective study to investigate the feasibility and safety of acupuncture treatment on taxane-induced peripheral neuropathy and QoL in Korean breast cancer patients. Our results provide important data to apply acupuncture as a useful alternative to the treatment of CIPN in breast cancer patients.

## 5. Conclusions

Acupuncture improved symptoms of CIPN and QoL in Korean women suffering from peripheral neuropathy after chemotherapy for breast cancer. The effects of acupuncture lasted for at least 1 month after the treatment. Acupuncture can be a useful and safe alternative for the treatment of CIPN in breast cancer patients. A randomized, controlled prospective study with a larger sample size and long-term follow-up is required to verify the role of acupuncture in the management of CIPN in patients with breast cancer.

## Figures and Tables

**Table 1:** Acupuncture points prescriptions and their anatomical position.

Prescription			Location
Name (English/Han Geul)	Point	Meridian	
Joining Valley/hap gok	LI 4	Large intestine meridian	On the dorsum of the hand, between 1^st^ and 2^nd^ metacarpal bones, in the middle of the 2^nd^ metacarpal bone on the radial side

Pool at the Crook/gok ji	LI 11	Large intestine meridian	At the lateral end of the transverse cubital crease midway between the cubital crease on the radial side of the biceps brachii tendon and the lateral epicondyle of the humerus

Leg Three Miles/jok sam ni	ST 36	Stomach meridian	3 cun^a^ below ST 35^b^, one finger width lateral from the anterior border of the tibia

Middle Margin/tae chung	LV 3	Liver meridian	On the dorsum of the foot, in the depression proximal to the 1^st^ metatarsal space

Eight Evils/pal sa	M-UE 9 (*Ba Xie*)	Extra points	On the dorsum of the hand, at the webs between each finger, at the junction of the red and white skin

Eight Winds/pal poong	M-LE 8 (*Ba Feng*)	Extra points	On the dorsum of the foot between the web and metatarsophalangeal joint

^a^3 cun, the width of all four fingers except the thumb, measured at the level of the proximal interphalangeal joint of the index finger.

^b^ST 35, with knee flexed, below the patella in a depression lateral to the patellar ligament.

^c^SP 5, a point in a depression distal and inferior to the medial malleolus midway between the tuberosity of the navicular bone and the tip of the medial malleolus.

^d^ST 41, a point on the midpoint of the transverse crease of the ankle, approximately level with the tip of the external malleolus, in a depression between the tendons of extensor digitorum longus and hallucis longus.

**Table 2:** Demographic and clinical characteristics of the participants.

Variable	N (%) or Mean ± SD
Age, years	58.7 ± 7.5

Height, cm	153.9 ± 5.3

Weight, kg	59.9 ± 7.5

Menopausal status	
Premenopause	1 (10)
Postmenopause	9 (90)

Comorbidity	
Yes	8 (80)
No	2 (20)

Type of surgery	
Mastectomy w/ axillary lymph node dissection	6 (60)
Breast conserving surgery w/axillary lymph node dissection	3 (30)
Breast conserving surgery w/sentinel node biopsy	1 (10)

Grade of CIPN	
Grade I	4 (40)
Grade II	5 (50)
Grade III	1 (10)
Grade IV	0 (0)

SD, standard deviation; CIPN, chemotherapy induced peripheral neuropathy.

**Table 3:** Results of nerve conduction study from baseline to the end of the final acupuncture session.

Participants	Findings of nerve conduction study		
	Baseline	At the end of the last treatment	Results
1	No electrophysiological evidence^a^ of peripheral neuropathy in all extremities	No electrophysiological evidence^a^ of peripheral neuropathy in all extremities	Normal

2	Suggestive of sensory polyneuropathy^b^ in all extremities	Suggestive of sensory polyneuropathy^b^ in upper and lower extremities	No interval change

3	Suggestive of right distal median neuropathy	No electrophysiological evidence^a^ of peripheral neuropathy in all extremities	Improved

4	Suggestive of sensory polyneuropathy^b^ in all extremities	Suggestive of right distal median neuropathy	Improved

5	Suggestive of sensory polyneuropathy^b^ in all extremities	Suggestive of sensory polyneuropathy^b^	No interval change

6	Suggestive of sensory polyneuropathy^b^ in lower extremities	Suggestive of sensory polyneuropathy^b^ in lower extremities	No interval change

7	Suggestive of sensory polyneuropathy^b^	Suggestive of sensory polyneuropathy^b^	No interval change

8	No definite electro-physiologic evidence^a^ of neuropathy	No definite electro-physiologic evidence^a^ of neuropathy	Normal

9	Indicative of left median neuropathy	Suggest left median neuropathy	Improved

10	No electrophysiological evidence^a^ of polyneuropathy, radiculopathy, myopathy	No definite electro-physiologic evidence^a^ of polyneuropathy	Normal

^a^No electrophysiological evidence, normal nerve conduction studies (NCS) including motor NCS, sensory NCS, F wave study, and H-reflex study.

^b^Suggestive of sensory polyneuropathy, decreased sensory nerve action potential amplitude and sensory nerve conduction velocity with normal motor NCS, F wave, and H-reflex study.

**Table 4:** Changes of quality of life of breast cancer patients with CIPN assessed by the SF-36 from baseline to the end of the final acupuncture session.

Variable	SF-36 score, mean (SD)		F (*p*-value)
	Baseline	At the end of the last treatment	
Physical functioning	70.00 (22.236)	77.50 (17.361)	5.548 (0.043)^∗^

Role limitations due to physical health problems	54.38 (22.831)	69.38 (20.927)	11.676 (0.008)^∗^

Role limitations due to emotional problems	58.33 (36.430)	71.67 (21.943)	1.595 (0.238)

Vitality (energy/fatigue)	48.13 (28.573)	51.88 (21.661)	0.503 (0.496)

Emotional well-being (general mental health)	55.50 (16.406)	64.50 (18.626)	4.734 (0.058)

Social functioning	60.00 (28.747)	82.50 (18.819)	12.356 (0.007)^∗^

Bodily pain	47.50 (19.149)	127.25 (228.207)	1.258 (0.291)

General health perceptions	50.00 (26.667)	58.00 (26.479)	16.000 (0.003)^∗^

^*∗*^Indicates statistically significant (*p* < 0.05).

**Table 5:** Summary of the studies investigating the use of for the treatment of CIPN from the literature.

Study	Design of the study	Number of patients	Included cancer types	Intervention and control	Protocol of acupuncture	Acupoint	Outcome measurement	Effects of acupuncture/Other results
Shiqiang et al., 2017 [11]	Prospective, randomized, single-blind	27	Stomach, intestine, lung, ovary, breast	Acupuncture vs EA	Two courses of treatment: once per day for 7 days (starting at the day before chemotherapy) then 14 days off, for total of 21 days per course	LI4, LV3	PN, clinical symptoms, QoL, immune function	Improved/EA better than acupuncture

Donald et al., 2011 [12]	Retrospective, case series	18	MM, ALL, CML, colon, caecum, breast, ovary	Acupuncture, no control	Once a week for 6 weeks	SP6, ST36, LV3, LI4, BL60, *Ba Feng, Ba Xie*	Subjective symptoms	Improved (82%)

Schroeder et al., 2012 [13]	Prospective, non-randomized, non-blinded, pilot study	11	Breast, colon, bronchial, lymphoma	Acupuncture and best medical care vs best medical care	for 10 weeks	ST34, EX-LE12, *Ba Feng*	NCS	Improved/Acupuncture better than control

Wong et al., 2006 [18]	Prospective case series	5	Gynecological cancer	Acupuncture, no control	Two courses of treatment: once a week for 6 weeks, then 4 weeks off, for total of 16 weeks	CV6, ST36, LI11, *Ba Feng, Ba Xie*	Pain score, WHO CIPN grade	Improved

Ben-Horin et al., 2017 [26]	Retrospective analysis	30	Breast	Acupuncture and Reflexology (joint protocol)	1 to 2 weekly, no available duration	LU11, LI1, PC9, TE1, HT9, SI1, SP1, LR1, ST45, GB44, BL67, KI1	Symptom severity assessed by physician	Improved/Symptom-free at 6 months after starting treatment

Xiong et al., 2016 [27]	Prospective, randomized	90	Breast	Acupuncture vs mecobalamin injection vs acupoint injection	Once every 3 days for 10 times	LI11, LI4, ST36, SP6, SP10	NCS, hemorrheology indicators	Improved/Acupoint injection with mecobalamin better than acupuncture

Bao et al., 2011 [30]	Case report	1	MM	Acupunctur, no control	Once per week for 6 treatments, then every other week for 4 treatments, then once every three weeks for 2 treatments, then once per month for 2 treatments, for total 14 acupuncture treatments	Shen men, point zero, two additional auricular acupuncture point, LI4, SJ5, LI11, ST40, *Ba Feng*	VAS pain score	Improved

Jeong et al. (this study)	Prospective, single arm, pilot study	10	Breast	Acupuncture, no control	Three times a week for 4 weeks	LI4, LI11, ST36, LV3, *Ba Xie*, *Ba Feng*	NPSI, NCS, SF-36	Improved

CIPN, chemotherapy induced peripheral neuropathy; EA, electroacupuncture; PN, peripheral neuropathy; QoL, quality of life; MM, multiple myeloma; ALL, acute lymphoblastic lymphoma; CML, chronic myeloid lymphoma; WHO, World Health Organization; NCS, nerve conduction study; VAS, visual analogue scale; NPSI, Neuropathic Pain Symptom Inventory; SF-36, 36-Item Short From Health Survey.

## Data Availability

Due to ethical concerns, supporting data cannot be made openly available. Further information about the data and conditions for access are available at the Comprehensive and Integrative Medicine Institute data archive. Please contact yjjeong@cu.ac.kr for data access.
